# Ultra-Low Intensity Post-Pulse Affects Cellular Responses Caused by Nanosecond Pulsed Electric Fields

**DOI:** 10.3390/bioengineering10091069

**Published:** 2023-09-10

**Authors:** Kamal Asadipour, Carol Zhou, Vincent Yi, Stephen J. Beebe, Shu Xiao

**Affiliations:** 1 Department of Electrical and Computer Engineering, Old Dominion University, Norfolk, VA 23529, USA; kasad001@odu.edu; 2 Frank Reidy Research Center for Bioelectrics, Old Dominion University, Norfolk, VA 23529, USA; czhou@odu.edu (C.Z.); sbeebe@odu.edu (S.J.B.); 3 Ocean Lakes High School, Virginia Beach, VA 23454, USA; vincentyi98@gmail.com

**Keywords:** nanosecond pulse, post-pulse, charging current, intracellular effects, spare respiratory capacity

## Abstract

High-intensity nanosecond pulse electric fields (nsPEF) can preferentially induce various effects, most notably regulated cell death and tumor elimination. These effects have almost exclusively been shown to be associated with nsPEF waveforms defined by pulse duration, rise time, amplitude (electric field), and pulse number. Other factors, such as low-intensity post-pulse waveform, have been completely overlooked. In this study, we show that post-pulse waveforms can alter the cell responses produced by the primary pulse waveform and can even elicit unique cellular responses, despite the primary pulse waveform being nearly identical. We employed two commonly used pulse generator designs, namely the Blumlein line (BL) and the pulse forming line (PFL), both featuring nearly identical 100 ns pulse durations, to investigate various cellular effects. Although the primary pulse waveforms were nearly identical in electric field and frequency distribution, the post-pulses differed between the two designs. The BL’s post-pulse was relatively long-lasting (~50 µs) and had an opposite polarity to the main pulse, whereas the PFL’s post-pulse was much shorter (~2 µs) and had the same polarity as the main pulse. Both post-pulse amplitudes were less than 5% of the main pulse, but the different post-pulses caused distinctly different cellular responses. The thresholds for dissipation of the mitochondrial membrane potential, loss of viability, and increase in plasma membrane PI permeability all occurred at lower pulsing numbers for the PFL than the BL, while mitochondrial reactive oxygen species generation occurred at similar pulsing numbers for both pulser designs. The PFL decreased spare respiratory capacity (SRC), whereas the BL increased SRC. Only the PFL caused a biphasic effect on trans-plasma membrane electron transport (tPMET). These studies demonstrate, for the first time, that conditions resulting from low post-pulse intensity charging have a significant impact on cell responses and should be considered when comparing the results from similar pulse waveforms.

## 1. Introduction

In recent years, there have been significant advancements in the field of bioelectrics, specifically in the study of nanosecond pulsed power technology and its effects on cellular responses [1,2,3]. Intense nanosecond pulses have been shown to induce diverse biological responses, such as membrane permeabilization [4], DNA damage, and activation of signaling pathways [5,6]. Nanosecond pulsed electric fields (nsPEFs) have emerged as a promising tool for various biomedical applications such as tissue treatment [7,8], atrium ablation for heart defibrillation [9,10], and immune response expression for cancer treatment [11,12,13,14].

Nevertheless, different research groups have used custom-designed and custom-manufactured pulse generators, resulting in varying pulse conditions [15,16,17,18,19,20,21,22,23,24,25,26]. This may present a perplexing and challenging situation when comparing and interpreting results. Results of experiments involving intense nanosecond pulses typically report the electric field, pulse duration, and pulse number, but nuances in pulse waveforms such as rise time, pulse plateau, and fall time also exist [27,28]. Even the pulse plateau is not perfectly flat and can rise or fall to a certain degree between the rise time and fall time. However, these waveform characteristics are often not well-characterized and not reported. The pulse rise time and fall time, for example, has been shown to affect mitochondrial membrane potential and cell viability under the assumption that a pulse rising faster can reach the cell interior more effectively bypassing the capacitive barrier of a cell membrane compared to a pulse rising slower [27]. The phenomenon of nanosecond bipolar cancellation (NBC) occurs when an additional pulse of opposite polarity is applied, resulting in weaker responses than the unipolar pulse condition where no such cancellation occurs [29,30,31]. This difference in results can be explained by the disruption of cell membrane charging by the opposite current before any harm to the cell is done. However, this is only one possible hypothesis, and other mechanisms may also be valid. Although standardizing pulse parameters is difficult, it is becoming evident that the pulse waveform details should be considered when interpreting results that are generated in close conditions.

As such, there is also a possibility that the charging current of a pulse generator (in the form of a post-pulse) could affect biological responses, but it is often overlooked after the main pulse due to its low intensity. The post-pulse can have a magnitude no more than 10% of the main pulse and often does not show up on the oscilloscope because of the small scale, while the biological response is solely attributed to the main pulse.

Our study, for the first time, investigated the effect of these post-pulses on biological responses elicited by nanosecond pulses. We used two pulse generators based on transmission lines, namely a pulse forming line (PFL) and a Blumlein line (BL), to demonstrate the different post-pulse characteristics while maintaining nearly identical main pulse features. It is worth noting that PFL and BL remain robust pulsers for in-vitro applications that require high current, low impedance, and short pulse duration (≤100 ns), despite the growing utilization of solid-state pulse generators [32].

Understanding the interplay between nsPEFs and ultra-low intensity post-pulses is crucial for advancing the applications of pulse power technologies in diverse fields. These findings may have implications for improving therapeutic strategies such as atrium ablation for heart defibrillation and enhancing immune response expression for cancer treatment. Moreover, unraveling the underlying mechanisms can provide valuable insights into the fundamental principles governing cellular responses to pulsed electric fields. Our study highlights the importance of characterizing and reporting pulse waveforms to enhance the reproducibility and comparability of results across different research groups using different pulse generators.

## 2. Results

### 2.1. BL Had a Low-Intensity Post-Pulse Opposite to the Main Pulse, Contrary to PFL, despite Having the Same Main Pulse

**Figure 1:**
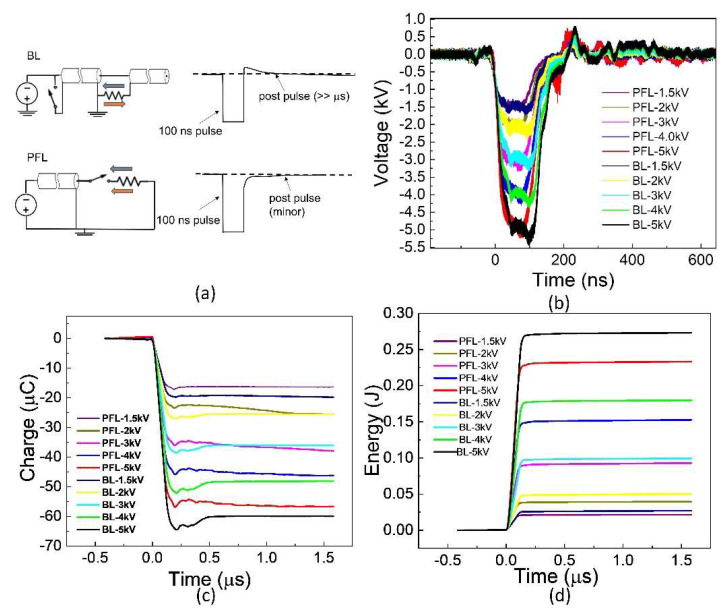
The waveforms were generated using two pulse generators: the Blumlein line (BL) and the pulse forming line (PFL). (**a**): The BL exhibits an opposite polarity post-pulse compared to the main pulse, whereas the PFL has a post-pulse with the same polarity. In the figure, the BL pulse is intentionally inverted to match the text, although it should be positive due to the negative charging power supply concerning the ground. The grey arrow: is pulse current; The orange arrow: post pulse current. (**b**): The waveforms display a voltage increase from −1.5 kV to −5 kV. Each waveform represents the average of 30 consecutive waveforms. (**c**): The charges delivered to the load are calculated by integrating the current over time. In this case, the load was a cuvette. (**d**): The energy deposited into the load.

The 100 ns pulses generated by PFL and BL are shown in Figure 1. These waveforms were obtained for the cuvettes with a resistance of approximately 10 Ω, which was needed to match PFL and BL. Five voltages (1.5, 2, 3, 4, and 5 kV) and ten waveforms are shown, with each representing the average waveform over 30 pulses with standard errors ±0.5 kV. For a given voltage, the rise time for PFL was slightly faster than BL, and the pulse duration for BL was slightly longer than that of PFL (<10 ns, measured at the full width at half maximum). The peak voltages of BL were slightly larger than those of PFL (<0.5 kV). The charges flowing through the load were calculated as the time integral of the voltage divided by the resistance, ∫VRdt, where R = 10 Ω (Figure 1c). The charge for BL was always slightly higher if not equal to that of PFL. Also shown in Figure 1d is the energy calculated by the integral of the power, ∫V2Rdt. As the voltage increased, the difference in energy deposited in the load resistance between PFL and BL became larger. However, the energy for BL was always larger than that of PFL.

**Figure 2:**
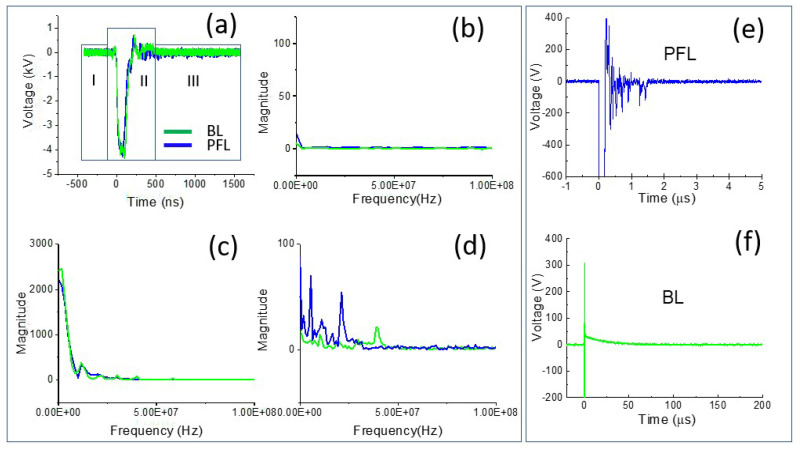
The pulse waveforms were analyzed in both the time and frequency domains. (**a**) The intervals of interest in the waveform, including the prepulse (−450 ns to −100 ns), main pulse (−100 ns to 500 ns), and post-pulse (500 ns to 1600 ns); the spectrum of the pulses was calculated for each interval using FFT: (**b**) the prepulse; (**c**) the main pulse; and (**d**) the post-pulse; (**e**,**f**) zoomed-in views of the post-pulses for both PFL and BL on a smaller voltage and longer time scale.

**Figure 3:**
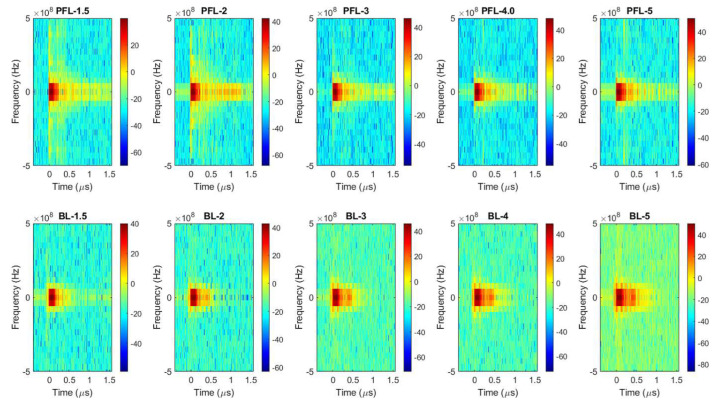
The spectrums of the pulses over time were calculated for the PFL and BL waveforms using STFT performed on the data shown in Figure 1b. **Top** row: the PFL voltages (−1.5 kV to −5 kV); **bottom** row: the BL voltages (−1.5 kV to −5 kV). The color bars show the magnitude of the spectrum.

To examine the difference in the spectrum of the PFL and BL pulses, Fourier transform (FT) was performed on the 4 kV data (both PFL and BL) over three time-intervals: the prepulse (−450 ns to −100 ns), the main pulse (−100 ns to 500 ns), and the post-pulse (500 ns to 1600 ns) (Figure 2a–d). In the prepulse interval, no signal was observed (Figure 2b). In the main pulse interval, the BL spectrum almost overlapped with that of the PFL, although it appeared slightly higher in the near DC frequency (Figure 2c). In the post-pulse interval, the PFL spectrum appeared higher than the BL one near the low frequencies (up to 2.5 × 10^7^ Hz). Furthermore, the post-pulse difference between the PFL and BL waveforms for all voltages can be observed in Figure 3, obtained using STFT (short-time Fourier Transform). The PFL post-pulses consistently exhibited a more extended signal spread than the BL pulses, despite mostly being low intensity.

Such a large difference led us to re-examine the post-pulse phases in the time domain, but this time at a much smaller voltage scale and longer time. Figure 2e,f show the post-pulses for both PFL and BL. The main pulses were both −4 kV but were truncated to highlight the difference. In the case of PFL, the post-pulse had the same polarity as the main pulse and lasted for less than 2 µs. On the other hand, for the BL, the post-pulse was much longer (50 µs) but had the opposite polarity to the main pulse. It is worth noting that both BL and PFL’s post-pulses consisted of two components: the charging voltage and a mismatched component resulting from the slight impedance mismatching between the transmission line and the cuvette. In terms of duration by excluding the mismatched component, the charging pulses extended much longer, although their magnitudes were small and decaying: the PFL’s post-pulse was (5%) of the main pulse, whereas the BL’s was (1–2%) of the main pulse.

### 2.2. PFL Pulses Extended the Duration of OMP and Posed Less Change on IMP than BL Pulses

**Figure 4:**
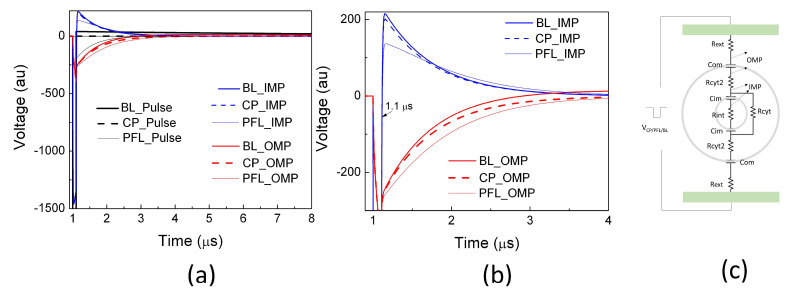
The potential drops were simulated using a linear equivalent cell model by applying a clean pulse (CP), a PFL pulse, and a BL pulse at 1 μs. The potential between the outer membrane (OMP) and the potential between an intracellular organelle (e.g., mitochondrion) (IMP) are shown in (**a**) on a larger scale (both in voltage and time) and (**b**) on a smaller scale. (**c**) The equivalent cell model in Pspice (Version 9.1) along with the parameters (R_ext_= 1 kΩ, C_om_ = 100 pF, R_cyt2_ = 100 Ω, C_im_ = 10 pF, R_cyt_ = 10 kΩ) [33].

Using a linear cell model consisting of resistances and capacitances representing the cell structure [33], potential drops across the outer membrane (OMP) and intracellular organelle membrane (IMP) were calculated for three scenarios: the clean pulse, the PFL pulse, and the BL pulse. During the 100 ns main pulse, there was no discernible change in the OMP among all pulse conditions. Moreover, the pulses in all cases resulted in approximately a threefold increase in the IMP compared to the OMP, indicating that nanosecond pulses generally bypass the outer membrane and penetrate to the cytoplasm for intracellular manipulation (Figure 4). After the main pulse (>100 ns), the IMPs reversed their polarity and swung in the opposite direction, with the magnitude being 13.3% of that during the main pulse for the CP and BL pulses. Conversely, the PFL post-pulse caused a smaller change in the IMP, reducing it to only 8% of its value during the main pulse. Additionally, the PL post-pulse sustained the OMP longer than the CP and BL pulses. The BL pulse dissipated the OMP rather rapidly (<4 μs) and led to a reversed OMP.

The ability of the BL post-pulse to reverse the OMP is significant due to its much longer duration (>100 ns), despite maintaining a low voltage. Generally, the PFL post-pulse, which shares the same polarity as the main pulse, prolongs the duration of the OMP, while the BL post-pulse shortens and even reverses the OMP. Moreover, the PFL post-pulse induces less change in the IMP compared to the BL post-pulse. These observations suggest that the PFL pulse can sustain membrane potential changes in both IMP and OMP for a longer duration compared to the BL pulse, indicating its potential for greater effectiveness in induing cellular responses. However, it should be noted that this model has limitations, as it assumes intact cell membranes and constant resistances without considering factors such as electroporation, cell shapes, orientations, etc. It thus provides a qualitative analysis that predicts the general trend of the potential changes resulting from electric pulses but does not reflect the absolute membrane potential changes.

### 2.3. Effects of PFL and BL Pulsers on Cellular Plasma Membrane Responses

Plasma membranes (PMs) are best known as physical barriers that define the cell and maintain ion transport across the membrane as a means of excitability and homeostatic maintenance. The PM also exhibits an electron transport (ET) mechanism carried out by plasma membrane redox systems (PMRSs). These ET systems transfer electrons from either intra- or extracellular donors to extracellular acceptors [34,35]. They regulate cellular redox homeostasis by maintaining the NAD(P)+/NAD(P)H ratios and attenuate oxidative stress acting as a compensatory mechanism during the stress, and aging process [36]. Given the known effects of electric fields to electroporate the PM, it was of interest to see if the PFL and BL pulsers had different effects on PM permeabilization as propidium iodide uptake and effects on trans plasma membrane electron transport (tPMET). 

**Figure 5:**
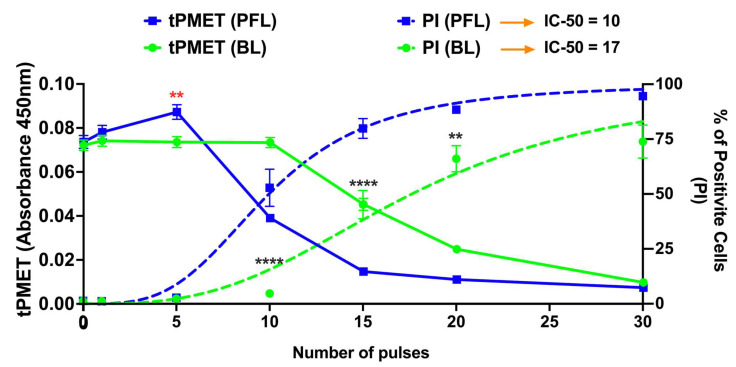
nsPEF effects of BL and PFL pulsers on tPMET and PI uptake. The tPMET rates defined as the rate of increase in WST-8 absorbance per min of reaction (left axis, solid lines), and PI fluorescence (Right axis, doted lines) were determined by plate reader (10–35 min) and flow cytometry (5 min) respectively in a different assay. B16F10 cells were exposed to different pulsing numbers with BL or PFL (green and blue color code respectively) with a fixed electric field of 40 kV/cm. BL pulser showed the inhibitory effect on tPMET (significant decrease start at 20 pulses compared to control) while the PFL showed the biphasic effect on tPMET with a significant increase at fewer pulsing numbers (5 pulses, showed by red **) and then decrease for high pulsing number (significant decrease at 10 pulses). Significant differences were observed between these two pulsers in regard to an increase in PI uptake (at 10, 15, 20, and 30 Pulses), indicated by the (****). (*n* = 3) ** *p* < 0.05 and **** *p* < 0.0001.

Figure 5 shows two distinct responses from the plasma membrane activity of the PMRS regulating tPMET rates and plasma membrane permeability to propidium iodide (PI) in response to the PFL and BL pulsers. The linear tPMET velocity rates were measured in the 10–35 min range, which serves to measure the tPMET activity of the PMRS in B16F10 cells after pulsing with the BL or PFL pulser. The PFL pulser showed biphasic tPMET rates across different ranges of nsPEF pulsing. Under lower pulsing conditions (≤5 pulses), nsPEFs increased tPMET rates above the control rates while there was no increase in PI influx. However, under higher pulsing conditions (≥10 pulses), tPMET rates decreased below the control rates as there were increases in PI influx coincident with the decrease in tPMET. The maximum dimension of the PI molecule is typically 1.4 nm. Therefore, the absence of PI uptake does not definitively prove that the cell membrane is completely electroporation pore-free, as nsPEFs have been observed to create smaller nanopores that can cause Ca^2+^ influx [37]. In contrast, the BL pulser at low pulsing conditions (≤5 pulses) showed the same level of tPMET activity as the control. However, as the pulse number was increased to ≥20, a significant pulse number–dependent reduction in tPMET activity was observed coincident with a pulse number-dependent increase in PI influx. In general, for both the decrease in tPMET and the increase in PI influx, the PFL has a lower threshold or is more sensitive for determining these changes in cell responses. Thus, the PFL pulses can elicit a biphasic response, stimulating tPMET activity with a low number of pulses, but inhibiting it with a high number of pulses. In contrast, the BL pulses did not induce such a biphasic response and only inhibited tPMET.

### 2.4. PFL Has a Lower IC 50 for Cell Death Induction than BL

**Figure 6:**
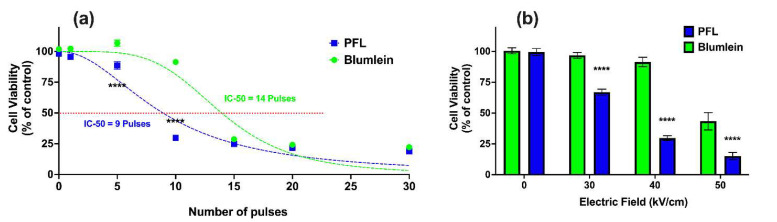
nsPEF effects of BL and PFL pulsers cell viability. Cell viability of B16F10 cells was determined using a plate reader after 24 h for (**a**) various pulsing numbers with a fixed electric field of 40 kV/cm, or (**b**) different electric fields (0, 30, 40, and 50 kV/cm) of 10 pulses, with BL (green) or PFL (blue) pulsers. In (**a**), significant differences were observed between these two pulsers, particularly at 5 and 10 pulses. In (**b**), the viability did not show a significant decrease compared to the control at 30 and 40 kV/cm with BL pulsing, whereas with PFL pulsing, a significant decrease in viability was observed (**** *p* < 0.0001).

Figure 6a shows the effects on cell viability 24 h after PFL and BL pulsing (100 ns, 40 kV/cm) with different pulsing numbers. Viability was found to be dependent on the number of pulses, such that the PFL IC_50_ value was 9 pulses and the BL IC_50_ value was 14 pulses. For 100 ns and 40 kV/cm, the decrease in cell viability from 95% to 25% occurred between 5 and 15 pulses for the PFL pulser and between 10 and 20 pulses for the BL pulser.

Figure 6b considers the electric field decreases in cell viability with the 100 ns pulses at 10 pulses. For PFL pulses, cell viability began to decrease at a threshold of 30 kV/cm. On the other hand, a significant decrease in viability for BL pulses was observed only when the electric field was raised to 50 kV/cm, with a slight decrease noticeable at 40 kV/cm. 

### 2.5. Differential Loss of ΔΨm with Increases in mROS Production with the PFL and the BL Pulsers

**Figure 7:**
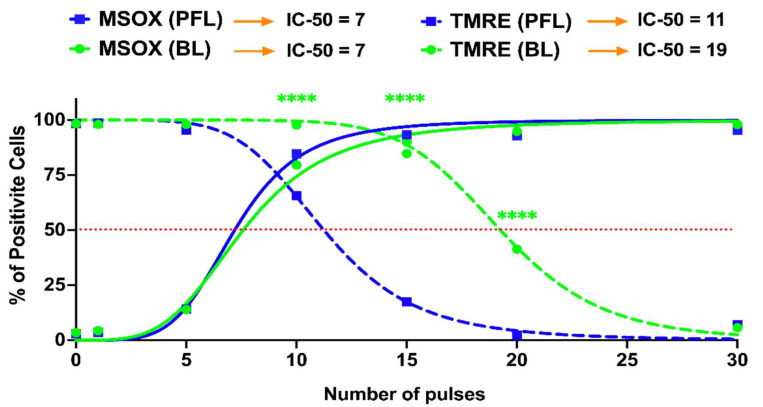
nsPEF effects of BL and PFL pulsers on the reactive oxygen species and mitochondria membrane potential at 20 min after pulsing. B16F10 cells were exposed to different pulsing numbers with BL or PFL (green and blue color code respectively) with a fixed electric field of 40 kV/cm. Dotted lines represent the TMRE and solid lines represent the MSOX. The IC-50 is mentioned at the top. Significant differences were observed between these two pulsers in regard to a decrease in mitochondrial membrane potential (at 10, 15, and 20 pulses), indicated by the (**** with *p* < 0.0001).

Figure 7 shows nsPEF-induced mitochondrial ROS (mROS) production determined by MitoSox (solid lines) and change in the ΔΨm (dotted lines) as pulse numbers are increased at 40 kV/cm. In contrast to the differential loss of ΔΨm as the PFL (blue lines) and BL pulse (green lines) numbers increased, there was no difference in the production of ROS between the two pulsers. For the PFL, 75% of cells were mROS positive and only about 25% of cells had a loss in ΔΨm. In contrast, essentially all the cells were mROS positive before there is a significant loss in loss in BL ΔΨm. The losses in ΔΨm were nearly parallel with 50% of cells showing a loss in ΔΨm for the PFL and BL at about 12 pulses 20 pulses. Thus, the PFL was more sensitive than BL for loss of ΔΨm but there were no differences between the two pulsers in pulse number for mROS production. The difference in the response thresholds of ΔΨm and ROS indicates that the ROS mechanism is not directly linked to the ΔΨm mechanism. This disparity in response solely attributable to the pulse condition is noteworthy.

### 2.6. PFL but Not BL Caused a Decrease in Maximal OCR and Spare Respiratory Capacity (SRC)

**Figure 8:**
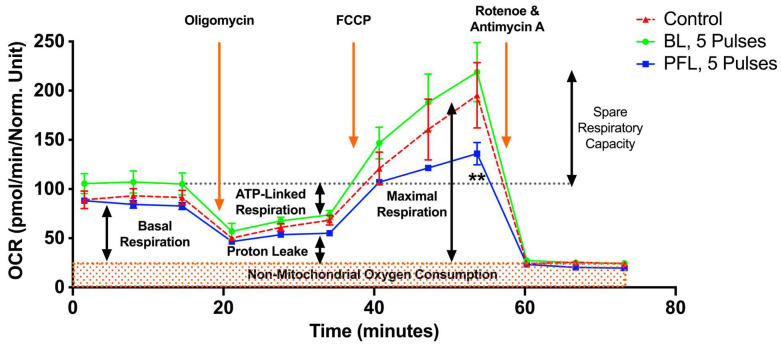
nsPEF effects of BL and PFL pulsers on mitochondrial oxidative metabolism in B16F10 melanoma cell lines. The oxygen consumption rate (OCR) of cells was measured 15 h after pulsing with 5 pulses. The x-axis represents time (up to 75 min), which aligns with the recommended test profile in the Seahorse assay for measuring mitochondrial respiration. The electric field was maintained at 40 kV/cm for both pulsers. The cells were maintained at 37 °C during the 15 h while they adhered. The different states of mitochondrial respiration are indicated: basal respiration (Basal), proton leak (respiration after oligomycin exposure), maximal respiratory capacity (respiration after FCCP, MRC), and non-mitochondrial respiration (after rotenone and antimycin A) (NM). * *p* < 0.05 compared to control. Cells treated with PFL pulses showed a lower SRC compared to the control group (** *p* < 0.002).

Figure 8 shows the metabolic effects of PFL and BL nsPEFs on oxygen consumption rate (OCR) using the Seahorse. Cells were treated with nsPEFs and then incubated until they were attached, as required for analyses. A look at responses that were measured after 5 pulses within the first 30 min after pulsing indicates that there was no ROS production or loss of ΔΨm (Figure 7), no PI uptake or no loss in tPMET (Figure 5), and no loss in viability after 24 h post pulse (Figure 6). However, for the PFL, there was an increase in tPMET. Seahorse results show that there was no significant decrease in basal OCR 15 h after nsPEFs with either pulser. However, after FCCP (uncoupling agent) treatment, the PFL treatment resulted in a significant decrease in maximal OCR and a decrease in spare respiratory capacity (SRC) determined by FCCP OCR minus basal OCR. BL pulses led to a slight but insignificant increase in SRC. The SRC reflects the mitochondria’s ability to fulfill additional energy requirements beyond the basal level in response to acute cellular stress. Thus, PFL pulsers show differences in responses to maximal OCR and SRC that are not present in the BL pulser and not present in basal conditions for either pulser occur 15 h after nsPEF treatment.

## 3. Discussion

These studies show that nanosecond pulses generated by commonly used pulse generators (PFL and BL) with the same pulse duration and essentially the same electric field and frequency distributions can result in different cell responses owing to distinct post-pulse waveforms determined by their dissimilar circuit topology. These subtle post-pulse waveform differences, which have been overlooked, can have a significant impact on functional outcomes. Specifically, the PFL post-pulse waveform was unipolar, while the BL pulse was bipolar. For instance, at 4 kV, the PFL pulse exhibited a small post-pulse waveform (5% of the main pulse, same polarity) lasting approximately 2 μs. Conversely, the BL pulse had an even smaller post-pulse waveform (1–2% of the main pulse, opposite polarity) but lasted longer (~50 μs). These post-pulses were a result of their electrical configurations being unique. In the PFL configuration, the load (cells in cuvette) was isolated from the charging circuit by a switch. After the switch closed, allowing the 100 ns pulse current flow, there was a brief charging current from the high voltage power supply. However, this current stopped quickly as the switch recovered and isolated the load from the charging circuit. The recovery process occurred on a scale of 2 μs, much shorter than that of a conventional spark gap switch [18]. This could be attributed to the small energy involved (100 mJ) and the short pulse duration (100 ns), whereas a conventional spark gap switch can handle >10 J and conduct for >1 ms. In our case, the discharge mode might involve a streamer-arc channel without significant heating of the ambient air, allowing for a rapid switch recovery. On the other hand, in the BL configuration, the load was continuously connected to the BL and remained in the charging loop regardless of the switch state. A small charging current was present throughout the charging time until the BL was fully charged before the next pulse (Figure 1a). 

**Figure 9:**
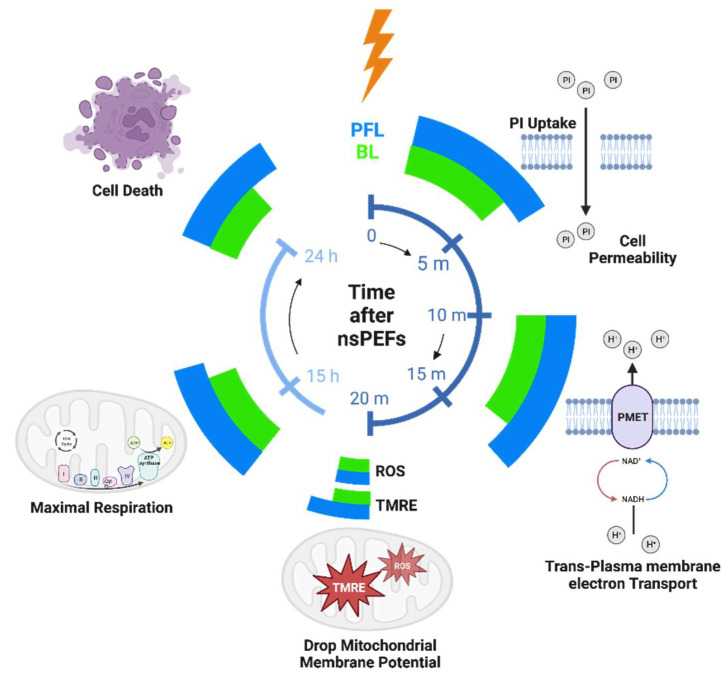
The timeline of the cell responses to PFL and BL pulses at different time intervals: 10–50 min, 15 h, and 24 h after pulsing. The magnitude of cell responses is represented by the extension of azimuthal angels (larger angle meaning larger response). Created with BioRender.com.

The distinction in cell responses to PFL pulses and BL pulses, as summarized in Figure 9, can be attributed to the differences in their post-pulse condition. During the main pulse interval, the frequency contents, charges, and energy of both types of pulses were almost identical, with some cases where the BL pulses exceeded the PFL pulses. However, in the post-pulse interval, compared to the BL post-pulses, the PFL post-pulses demonstrated a longer duration effect on the OMP and had a lesser impact on the IMP established by the main pulse, (Figure 4). Given that the MP created by the main pulse leads to membrane pore formation, Ca++ influx, and other effects, it would be expected to elicit a stronger cell response for holding longer. Therefore, it is not surprising that the PFL pulses generally demonstrated greater potency than the BL pulses.

The main pulse waveforms of PFL and BL, which are nearly identical in charge and spectrum, can induce similar membrane and intracellular effects. For example, the charging of the cell’s outer membrane can lead to an amplified electric field across it, resulting in pore formation and increased membrane permeability. However, the subsequent post-pulse current can modify the membrane potential by neutralizing the charges that have accumulated across the membrane. This effect is particularly pronounced in the case of BL pulses, which have an opposite post-pulse current. The charge of the main pulse was estimated as −40 μC (−4 kV × 100 ns/10 Ω), which is close to the measured value of −50 μC presented in Figure 1c. On the other hand, the charge flowing during the post-pulse can be calculated by integrating over the post-pulse waveform to be 75 μC. This accounts for the same magnitude of the charge of the main pulse charge, which would have reduced the membrane charging established by the main pulse, and it would certainly cause a significant change in both the OMP and the IMP, as indicated in Figure 4. In contrast, in the case of PFL pulses, the post-pulse current does not significantly alter the membrane potentials initiated by the main pulse. However, it is possible that the potential could be slightly larger due to the same polarity of the post-pulse, which serves to maintain the charging of the membranes.

However, in addition to plasma membrane electroporation as PI influx, nsPEFs also shows a unique electric field modulation of a well-known but seldomly discussed activity of the plasma membrane redox system (PMRS) function of tPMET, which plays a crucial role in safeguarding cells against intracellular oxidative stress, maintains redox balance, and regenerates NAD+ for glycolysis [36]. Notably, in contrast to the BL pulser showing only a decrease in nsPEF-induced tPMET, the effect of the PFL induced a biphasic effect with an increase in tPMET at lower electric field conditions before any PI influx appeared and an inhibition of tPMET at higher electric fields where PI influx demonstrated PM EP. So, the presence of PM pores was coincident with the loss of tPMET for both PFL and BL. Although coincidence is not an indication of the cause, it does raise the question of the relationship between nsPEF-induced PM permeabilization (pore formation) and tPMET. Nevertheless, the increase in tPMET appears to be independent of PI permeability. However, it is possible that molecules smaller than PI, such as Ca^2+^ could gain entry at lower nsPEF conditions through pores smaller than PI [27]. Overall, for effects on the PM, the PFL has a greater sensitivity or lower pulse number threshold for all three PM effects on the PI influx, gain and loss of tPMET activity.

Having seen these differences between the PFL and the BL pulsers, it was of interest to see the effects on cell viability. While effects depend on different factors, in all studies of nsPEFs no cell line or tumor type has shown resistances to nsPEF elimination. Two models have shown vaccine effects as vaccinations [13,38,39], meaning that tumor-free animals are resistant to regrowing the treated cancer again. In the viability studies, like that seen for the PM responses, the PFL had a lower IC_50_ value for viability than the BL pulser, as shown by requiring fewer pulses and requiring a lower electric field. This is interesting because all the nsPEF pulsers in those studies were BL constructions. Although the construction of a PFL for studies is less practical than the BL construction because of half-charging voltage output, it would be interesting to determine if a PFL pulser would require lower electric fields or fewer pulses for tumor elimination and be more effective for inducing immunity and vaccination.

Having shown that nsPEFs cause a dissipation of the mitochondrial membrane potential (ΔΨm) [27], we were curious to determine what caused this loss of ΔΨm. One obvious possibility was that like nsPEF effects on the plasma membrane, they could also permeabilize the inner mitochondrial membrane (IMM). However, another way that the nsPEFs could cause a loss of the ΔΨm, is through opening the mitochondrial permeability transition pore (mPTP). When we saw that the nsPEF-induced loss of ΔΨm was enhanced by Ca^2+^ [27], we considered that membrane permeabilization does not require Ca^2+^ and further that Ca^2+^ effects are essentially always mediated through a protein. Therefore, we hypothesized that nsPEF-induced loss of ΔΨm was not due to permeabilization of the IMM but more likely due to opening the mPTP, a hypothesis yet to be proved. Although the identity of the mPTP has been controversial, it has recently been proposed that the mPTP is a dimer of the F0F1 ATP synthase [40,41] and that Ca^2+^ binding to F-ATP synthase β subunit triggers the mitochondrial permeability transition [42]. This is consistent with the role of Ca^2+^ to enhance the dissipation of the ΔΨm.

ROS is a well-known activator of the mPTP and disturbances in Ca^2+^ and oxidative stress are tightly coupled for opening the mPTP. NsPEF induces the production of ROS with no distinctions between the PFL and the BL pulsers. These observations that nsPEF-induced ROS and that the elevation of ROS was enhanced in the presence of Ca^2+^ have heightened our attention to determining the roles of nsPEF-induced ROS in mPTP opening. Although there is no established direct role of ROS in opening the mPTP, ROS has effects that indirectly influence opening the mPTP. Many factors determine the probability for opening the mPTP including Ca^2+^, ΔΨm, and the redox state of mitochondrial components, which can be influenced by ROS [43]. Many SH reagents were among the strongest stimulators of permeability transition, so it was proposed that thiol groups on some protein(s) played roles in opening the mPTP [44]. It was proposed that the mPTP is modulated by the redox state of pyridine nucleotides and glutathione at two independent sites, one of which could be the adenine nucleotide translocase (ANT) [45]. Although the protein structure of the mPTP is still not defined, cyclophilin D (CypD) is a well-characterized regulator of the mPTP. CypD has also been shown to be redox regulated by forming an intramolecular disulfide with a conformational change playing a major role in cell necrosis by opening the mPTP acting as a redox-sensor protein in mitochondria [46].

In many of our studies, we have monitored the dissipation of the ΔΨm in response to nsPEFs in the presence of Ca^2+^ and ROS indicators, and antioxidants to determine roles for Ca^2+^ and ROS in ΔΨm. Figure 7 is one of those studies using TMRE to determine changes in the ΔΨm and MitoSox (MSOX) to monitor mitochondrial ROS (mROS) changes in response to the PFL and BL pulsers. Interestingly the results indicate that ROS plays different roles for the loss of ΔΨm depending on the pulser. The pulse number-dependent increase in ROS is essentially the same with both pulsers showing a significant increase in mROS between 5 and 10 pulses and a maximum at 15 pulses. In contrast, the loss of ΔΨm is different between the two pulsers. 

Therefore, the relationships between mROS and loss of ΔΨm are dissimilar between the two pulsers. What is similar between the two pulsers with the other cell responses is that compared to BL responses, PFL responses are more sensitive for dissipation of ΔΨm, loss of cell viability, PI permeability, activation of tPMET activity, and loss of tPMET activity. In contrast to all these cell responses, nsPEF-induced ROS production is the same for both PFL and BL pulsers. This suggests that the nsPEF-induced loss of ΔΨm is relatively independent of the production of ROS. 

In another approach for analyzing these pulsers on biological responses, we evaluated metabolic responses using the Seahorse to determine nsPEF effects on oxygen consumption rate (OCR) in control (sham-treated) and nsPEF-treated cells with 5 pulses from each of the PFL and BL pulsers. The 5-pulse condition did not cause any cell death, PI permeabilization, increase in ROS, loss of ΔΨm, or loss of tPMET activity with either pulser. However, the 5-pulse treatment did induce an increase in tPMET activity with the PFL but not the BL pulser. It should be noted that except for cell death, all these cell responses were determined within ≤30 min after treatment. Yet 15 h after treatment there were no significant differences in basal OCR with either pulser. With the increase in OCR after the addition of the FCCP uncoupling agent, the BL OCR was not significantly different than the control while the PFL treatment exhibited a significant decrease in OCR compared to the control and the BL response. The spare respiratory capacity (SRC) of the cells (FCCP minus basal OCR), was lightly increased with the BL pulser but significantly decreased in the PFL, which was due to the attenuated FCCP response and independent of the basal OCR. This suggests that there was a time-dependent deterioration of the status of the mitochondria presumably in response to cellular stress and the ability to fulfill additional energy requirements beyond the basal level in response to acute cellular stress. While there was not a significant increase in ROS in the 5-pulse condition, ROS likely increased during the 15 h time it took the nsPEF-treated cells to bind to the Seahorse plate for OCR analysis. Given that the increased ROS response was the same for both pulsers and only the response to the PFL showed a decreased SRC, any hypothesized increase in cellular ROS would be expected in the response to the PFL and not the BL. However, other stress response signaling pathways could have been activated that were not analyzed such as activation of the mitogen-activated protein kinase (MAPK) pathway and the nuclear factor kappa-light-chain-enhancer of activated NF-κB pathway, which could also have been activated during the 15 h post nsPEF exposure to the PFL pulser [47]. These MAPK-NFκB stress pathways could have caused a deterioration of mitochondrial SRC, but this would have occurred in the PFL but not the BL pulser. Given that the PFL induced more sensitive responses than the BL, this could have resulted in a selective response of these stress pathways from the PFL, like that for the increase in tPMET. However, these are speculations since we did not analyze these enzymatic stress responses. Nevertheless, the differences in the metabolic response to the PFL and BL pulses provide another example of selectivity for a biological response from different nsPEF post-pulse waveforms. 

We previously published a conformation that a fast or short pulse rise time of the primary pulse was an important feature for inducing intracellular effects [27]. We were not cognizant at that time about roles for a post pulse, yet both fast and slow rise time pulsers were based on PFL design. Those studies showed that faster rise times vs. slower rise times were more effects to dissipate the ΔΨm and induce cell death while effects for Ca^2+^ or PI influx through the plasma membrane were not dependent on the pulse rise time. The present studies show yet a different way that nsPEF waveforms can have different and selective effects on cell responses. As indicated here, these different or selective effects have nothing to do with the primary pulse like the rise time studies just discussed but are related to the effects the post-pulse waveforms have on the primary pulse. 

Regardless of the different primary pulse waveforms based on their rise times or the post-pulse waveforms based on dissimilar circuit topology, these results show that dissimilar nsPEF waveforms can have distinctive and possible selective biological outcomes that can determine cell fate. Given that nsPEFs produce ROS and ROS are endogenous signaling molecules, it is most likely that nsPEF waveforms at the lower pulse conditions will have a greater impact on physiological functions while higher pulse conditions will be more typical of pathological conditions or for regulated cell death mechanisms. It is also possible that these nsPEF waveforms will initiate other non-ROS cellular responses.

Furthermore, in Figure 8, the impact of 5 pulses on mitochondrial oxygen consumption is shown. The PFL pulser showed a significant decrease in spare respiratory capacity (SRC) by reducing maximal respiration without affecting the basal respiratory level. This observation indicates a disruption in the ETC and/or proton transport across the inner mitochondrial membrane [48]. These findings are consistent with the notion that glycolysis-derived pyruvate oxidation is involved in maintaining SRC levels, which supports the stimulating effect observed on tPMET (Figure 5). In contrast, the BL pulser led to high SRC levels, a characteristic often associated with cancer cells that are resistant to targeted agents. This can be attributed to the fact that the low pulse number (5 pulses) in this experiment was insufficient to cause significant pore formation and promote the loss of the ΔΨm.

There have been previous studies that involved the deliberate introduction of post-pulses to investigate cell responses. One phenomenon that has been observed is NBC [29,31,49], where a reversed-polarity nanosecond pulse can reduce the cell responses caused by a preceding ns pulse. The underlying mechanisms for NBC are still not fully understood and may involve assisted membrane discharge, a two-step process of charge transfer, an alternating reduction and oxidation mechanism, as well as cation diffusion reversal. These mechanisms are more pronounced when the second pulse is of similar magnitude as the first pulse. In another study [50], a double pulse strategy has been used for electroporation, where a high voltage short pulse is used for electroporation and a low voltage long pulse facilitates drug delivery through electrophoresis. Bipolar pulses with high frequency characteristics have also been employed for irreversible electroporation (HFIRE) [51,52], with the reversed polarity pulses used to suppress or remove muscle twitching by exploiting different time constants between electroporation and muscle excitation [53]. In these studies, the post-pulse to main pulse ratios were much larger compared to our study. For example, in BPC, the best cancellation efficiency was observed when the reversed pulse magnitude was 50% of the first pulse. In HFIRE, the first pulse was delivered at a higher amplitude than subsequent pulses, but it was common for the second pulse to be equal to the first phase. In the double pulse strategy, the second pulse was also 10% to 100% of the first pulse. In our study, the magnitude of the post-pulse was less than 5% of the main pulse and determined by dissimilar circuit topology differing between the two designs, yet it still resulted in disparate cell responses. This suggests that a mechanism like electrophoresis may be involved in cells responding to the post-pulse. Further investigation into the mechanisms, specifically in the realms of electrokinetics and bioelectrochemistry, may help elucidate the underlying processes that have often been overlooked in pulse engineering.

## 4. Conclusions

In the studies here, two different 100 ns pulses generators were used providing 100 ns pulse durations and electric fields of 40 kV/cm primary pulses with similar voltage and frequency wave distributions. One was a PFL with a post-pulse waveform having the same polarity as the primary pulse and the other was a BL pulses with a post-pulse waveform having an opposite polarity as the primary pulse. The cell responses obtained from these distinct pulse generators were determined from their post-pulse waveforms, not their primary waveforms. 

Cells exhibited greater sensitivity to the PFL than the BL pulser with lower pulse numbers or electric field intensities for inducing cell membrane permeability, dissipation of ΔΨm, a decrease in mitochondrial SRC, a biphasic effect on tPMET, and eventual cell death. This biphasic behavior holds significant implications for enhancing the efficacy of ablation procedures and potentially facilitating cellular differentiation in cancer therapy, ultimately leading to the prospect of in-situ vaccination. Interestingly, both pulse types demonstrate a similar dependence on pulse number in terms of ROS production. Despite the post-pulse having a magnitude of less than 5% of the main pulse and lasting for a longer duration (50 µs), its low intensity is still expected to decrease the membrane potential caused by the main pulse. To the best of our knowledge, this is the first time that charging current, which is reflected as a post-pulse, has been reported to have such a significant effect on cellular response. This work highlights the importance of considering the charging characteristics in pulse generator design and when comparing cell responses under similar pulse conditions.

## 5. Material and Methods

### 5.1. Experimental Conditions and Protocols

#### Pulse generators and Cell Exposure System

Two pulse generators were utilized in the experiments. The first generator employed a PFL comprised of five 50 Ω cables (RG-8) to generate 100 ns pulses. The second generator utilized a BL constructed with ten 50 Ω cables (RG-58), also producing 100 ns pulses (see details in Chapts. 15 & 16 in [1]). For both pulse generators, the lengths of the cables were determined based on a propagation length of 5 ns/m. Under ideally matched conditions, both loads required a resistance of 10 Ω. The switches for these generators were atmospheric pressure spark gaps. These spark gaps consisted of polished, plane-plane brass electrodes and would self-close once the voltage exceeded the breakdown threshold. During the experiment, the breakdown voltages of the two pulse generators were regulated by adjusting the gap distances of the corresponding spark switches. Both generators were powered by the same high voltage supply (Glassman, series EH, 60 kV). No extra charging resistor was employed throughout the experiments. The pulse repetition rate was set at 1 Hz, controlled by the current setting on the power supply. Standard electroporation cuvettes with a 1-mm gap distance were used for the experiments. The solution contained within the cuvettes resulted in a resistance that was close to 10 Ω, eliminating the need for additional resistance for impedance matching. The pulse waveforms were measured with a custom-made, calibrated, high precision resistor divider (1000:1). 

### 5.2. Cell Culture

The murine melanoma cell line B16F10 (ATCC^®^ CRL-6475TM) was used in this study. The cells were grown in a humidified incubator at 37 °C with 5% CO2 in the Dulbecco’s Modified Eagle Medium (DMEM) produced by ATCC (30-2002), supplemented with 10% fetal bovine serum (FBS) (ATCC, 30-2020) and 1% of penicillin-streptomycin (Sigma-Aldrich). B16F10 cells were harvested with 0.25% (*w*/*v*) Trypsin- 0.1% EDTA solution (Corning, MT25053CI). The cells were passaged no more than 20 times. Initial cell counts and viability were determined using a 0.4% trypan blue exclusion viability assay (Corning, 25900CI). Cells with greater than 95% viability were washed with PBS, centrifuged at 300 RCF for 5 min at room temperature, and resuspended at a concentration of 1 × 10^5^ cells/ 100 µL for nsPEF treatments. In all experiments, cell suspensions were added to 100 µL cuvettes (1-mm gap sterile electroporation cuvette, BioSmith, U-72001) and treated with a BL or PFL pulser in the culture medium with the conductivity of 1.18 S/m.

### 5.3. tPMET Rate Determination

The Cell Counting Kit-8 (CCK-8/WST-8-reducing NADH oxidoreductase activity, Dojindo, CK04-11) was used here to measure the trans-PM electron transport (t-PMET) of the plasma membrane redox system (PMRS). The quantification of the final electron acceptor (WST-8 reduction) was based on the change in absorption at 450 nm per minute of incubation. Cell suspensions with a concentration of 4 × 10^5^ cells/100 µL were added to cuvettes for treatment with different pulsers and varying numbers of pulses. Following the nsPEF treatment, the CCK-8 reagent was added (at a 1:1 volume ratio) and mixed immediately, and the cells were transferred to 384-well plates (Greiner Bio-One CELLSTAR plate, with cover, from VWR, 50051816) with 30 µL per well. Microplate readers (Spectra Max i3) were used to measure the absorbance at 450 nm at 37 °C for 0–90 min. The tPMET rates were determined based on linear time courses between 10 and 35 min, as specified in the statistics analysis section.

### 5.4. Cell Viability Analysis

The Cell Counting Kit 8 (CCK-8, Dojindo, Kumamoto, Japan) was used to measure cell viability. B16F10 cells were grown to 80% confluency, and then the cell concentration was adjusted to 1 × 10^6^ cells/mL for nsPEF treatment. Following the pulsing, 15,000 cells were seeded into 96-well plates (Corning Incorporated, Corning, NY, USA). The cells were cultured for 24 h, after which 10 μL of CCK-8 solution (1:10 *v*/*v*) was added to each well. Following an additional 1.5 h incubation, the optical density was measured at an absorbent of 450 nm using a microplate reader (ELx800; BioTek Instruments, Inc., VT, USA). The OD value was divided by the control value to calculate the relative cell survival rate (background values were subtracted).

### 5.5. Flow Cytometry

ΔΨm was detected using tetramethylrhodamine ethyl ester, perchlorate (TMRE; Immunochemistry Technologies LLC, Bloomington, MN, USA). B16F10 cells were harvested, counted, and resuspended following the previously described method. The samples were then treated with the BL and PFL pulsers, and TMRE was added to the cells at a concentration of 0.3 μM. The cells were incubated for 20 min, protected from light. Cells were not subjected to pre-incubation prior to pulsing, as we observed that this could adversely impact cell viability [54]. The optimal approach is to introduce the dye immediately after pulsing and incubate it for precisely 20 min.

The same procedure was employed to detect ROS using MitoSOX-Red (MSOX; Invitrogen, Molecular Probes, Inc., Eugene, OR, USA), albeit with a final concentration of 2 μM. Red fluorescence from TMRE and ROS was detected in separate experiments using the PE channel on a Miltenyi MacsQuant Analyzer 10 flow cytometer, as both molecules share the same excitation/emission characteristics.

To detect cell permeabilization, cells were exposed to nsPEFs, and Propidium Iodide (PI; Invitrogen, P3566) was added to a final concentration of 10 µg/mL immediately after pulsing. Cells were then analyzed by flow cytometry 10 min after nsPEF treatment using the FITC channel [27]. Untreated and/or unstained samples were used as negative controls for treatment and fluorescence, respectively, in all experimental groups. Data analysis was conducted using FlowJoTM Software (Windows) Version 10 (Ashland, OR: Becton, Dickinson, and Company; 2019).

### 5.6. Seahorse Assay

The OCR (oxygen consumption rate) was measured using an XF HS Mini Analyzer (Seahorse Bioscience). Following the pulsing treatment, B16F10 cells were seeded into XFp cell culture 8-well mini plates in duplicate at a density of 3 × 10^3^ cells/well. The cells were then cultured under standard conditions for 15 h. Before measurement, the medium was replaced with Seahorse XF Assay Media (Agilent, Santa Clara, CA, USA) with a pH of 7.4. The assay media was supplemented with 10-mM glucose, 2-mM L-glutamine, and 1-mM pyruvate. For the mitochondrial stress test, the following inhibitors were used at the indicated final concentrations: 1.5-μM oligomycin, 1-μM FCCP, and 0.5-μM rotenone–antimycin A. Two wells without cells were included to assess non-cellular oxygen consumption, and the value of non-cellular oxygen consumption was subtracted from the cellular OCR value. After completing the experiment, the OCR data were normalized to the number of cells.

### 5.7. Statistics Analysis

The tPMET data obtained from the 10–30-min time period was subjected to linear regression analysis using GraphPad Prism version 9 (GraphPad Software, San Diego, CA, USA). Statistical analyses comparing the tPMET rates of the samples to the control were conducted using one-way ANOVA. For the Seahorse data obtained from the XF HS Mini, analysis and normalization of the number of cells were performed using Agilent Seahorse Wave Desktop software (Agilent Technologies, USA). Flow cytometry analysis was carried out using FlowJo^TM^ Software Version 10 (Ashland, OR: Becton, Dickinson, and Company; 2019). All experiments were conducted at least three times, and the data were expressed as Mean ± Standard Error of the Mean (S.E.M.). Statistical analyses such as one-way or two-way ANOVA were performed using GraphPad Prism, with a significance level of *p* < 0.05.

## Data Availability

The data presented in this study are available on request from the corresponding author.
